# Quantitative assessment of normal middle deltoid muscle elasticity at various arm abduction using ultrasound shear wave elastography

**DOI:** 10.1038/s41598-021-92074-6

**Published:** 2021-06-14

**Authors:** Lei Wang, Xuanyan Guo, Li Tan, Qin Chen

**Affiliations:** 1 Department of Medical Ultrasound, Sichuan Provincial People’s Hospital, University of Electronic Science and Technology of China, No.32, West 2nd section, Yihuan road, Chengdu, 610072 Sichuan China; 2 Department of Medical Ultrasound, Chinese Academy of Sciences Sichuan Translational Medicine Research Hospital, Chengdu, 610072 China

**Keywords:** Medical imaging, Ultrasonography, Skeletal muscle

## Abstract

The objective of this study is to assess the change in the normal MD elasticity using shear wave elastography (SWE) through measuring the middle deltoid (MD) elasticity in healthy participants at various arm abduction (with bilateral arms at 0 degrees abduction and 90 degrees active abduction) and analyzing the factors affecting normal MD elasticity. Mean shear wave velocity (SWV) of the MD in healthy right-handed participants were evaluated using SWE at different arm abduction, and potential factors (gender, MD thickness, age, body mass index) affecting MD elasticity were analyzed. Different arm abduction positions of each participant were as follows: (i) 0° abduction of bilateral arm (L0° and R0°), (ii) 90° active abduction of bilateral arm (L90° and R90°). Mean SWV was significantly higher at L90° than L0°, higher at R90° than R0°, higher at R0° than L0°, and higher at R90° than L90° (all *P* < 0.0001). SWV was significantly higher in males at both L0° (*P* < 0.05) and R0° (*P* < 0.01) than in females. Neither MD thickness, age nor body mass index influenced MD elasticity. Reference ranges of normal MD elasticity were 2.4–3.1 m/s in males and 2.2–2.9 m/s in females at L0° and 2.5–3.3 m/s in males and 2.4–3.2 m/s in females at R0°, and were 4.9–6.7 m/s at L90°, 5.2–7.1 m/s at R90° for both males and females. SWE is a feasible technique to assess normal MD elasticity at various arm abduction. Our results suggest that normal MD elasticity at L0°, R0°, L90°, and R90° with SWE are different. Moreover, these reference ranges may serve as quantitative baseline measurements for assessment of normal MD elasticity in the future.

## Introduction

Deltoid muscle is a vital muscle in maintaining shoulder movement and stability. On the one hand, it plays an important role in the biomechanics of shoulders going through reverse shoulder arthroplasty^1,2^. The importance of the deltoid in obtaining satisfactory effects after reverse shoulder arthroplasty has been investigated from clinical observation^2,3^. Pre- and postoperative conditions of the deltoid muscle have been identified as a key factor affecting the outcome of the operation. On the other hand, many patients have been suffering from deltoid muscle disorders due to long-time and repeated active abduction of the shoulder. Nevertheless, both pre- and postoperative quality assessment of the deltoid muscle still remain challenging. Middle deltoid (MD), as a vital segment of deltoid, plays a great role in elevating the arm in the scapular plane and mainly acts on arm abduction. MD muscle has been known to transform its mechanical environment during shoulder elevation dramatically after reverse shoulder arthroplasty^1–3^. However, MD muscle is hard to be quantitatively assessed by traditional methods. Electromyography (EMG) is a popular method for evaluating muscle activity^4^. However, it cannot assess individual muscle activity due to the influence of electrical activity in adjacent muscles^5,6^. The isokinetic dynamometer and handheld dynamometer are two feasible methods to assess muscle force. Nevertheless, the measurements are complicated and lack of stabilization^7,8^. Hence, it is necessary to find a new mean for quantitatively evaluating the MD.

Shear wave elastography (SWE) as a new ultrasound-based elastography technique, quantitatively providing the biomechanics parameters such as muscle elasticity, has good repeatability and reproducibility^9–11^. Excellent reliability and feasibility for assessing the deltoid using SWE were reported^3,12,13^. This novel SWE methodology to quantify the mechanical properties of the MD has important clinical significance for reverse shoulder arthroplasty. It may provide helpful information of the MD mechanical properties during treatment of reverse shoulder arthroplasty. SWE can quantitatively evaluate muscle elasticity through shear wave velocity (SWV) and Young’s modulus^10–14^. The initial measurement with SWE is SWV, and then SWV is converted into Young’s modulus for every pixel. Furthermore, there are inaccuracies when converting SWV to Young’s modulus^11,13–15^. Thus, SWV can be a better index for quantitatively assessing MD muscle elasticity.

However, there is little research on MD elasticity at various arm abduction using SWE. Up to now, there has not been uniform standard for quantifying normal MD muscle elasticity. The muscle fibers of the MD are parallel and superficial, and the MD elasticity can be easily measured. For deltoid muscle pain, the quantitative assessment of MD muscle using SWE may be a simple and feasible method, which has certain clinical significance. Our hypothesis is that if the normal reference ranges of the MD elasticity can be set up, it may be helpful to provide a quantitative baseline measurement for normal MD elasticity.

The objective of this study is to assess the MD elasticity and preliminarily establish the normal reference ranges of it at 0° arm abduction and 90° arm active abduction for bilateral examination using SWE.

## Methods

### Study design

This study was performed in accordance with the Declaration of Helsinki and approved by the Sichuan Provincial People's Hospital of China Ethics Committee. Informed consent for the acquisition and analysis of imaging data was obtained from the participants before starting their examinations. Participants were recruited between September 2019 and March 2020. The inclusion criteria were as follows: (i) healthy adults (≥ 18 years old); (ii) all participants were right-handed; (iii) deltoid with grade 5 muscle strength (normal muscle strength) without shoulder pain; (iv) good compliance with examination. The exclusion criteria were as follows: (i) pregnancy in women; (ii) shoulder surgery or trauma; (iii) taking myorelaxants or drugs affecting muscle elasticity; (iv) a history of tumor, rheumatoid, immune, metabolic, endocrine diseases, or musculoskeletal diseases. All study participants with normal muscle strength were examined under the guidance of rehabilitation physicians.

### Examination by SWE

The instrument used to acquire SWE images of the MD elasticity was Aixplorer US system (SuperSonic Imagine, Aix-en-Provence, France), with an SL 10–2 linear probe operating at 2–10 MHz. The musculoskeletal mode was preset. The ultrasound probe was placed parallel to the muscle fiber orientation^15,16^. The tip of the transducer was covered with several millimeters of US gel and placed perpendicularly to the shoulder skin smoothly without applying any pressure on the skin^17^. All participants were prohibited from exercising for 72 h before the experiment. And they were instructed to stay completely rested for 20 min before the examination. The SWE examination was performed on all participants by two experienced sonographers (A and B) who received SWE training. They were blinded to each other’s results for the entire study. 20 participants were randomly selected for consistency analysis. These 20 participants were examined by sonographer B and then measured again by sonographer A on the second day. The 20 participants were recalled to be checked again by sonographer A after a 1-wk interval.

### Study protocol

Different arm abduction positions in this study were as follows: left and right arm 0° abduction (L0° and R0°), left and right arm 90° active abduction (L90° and R90°). Each participant was asked to stand straight with the head in neutral position. The examining time of all healthy participants were set between 8:00 a.m. and 11:00 a.m. The angle of arm active abduction was measured by a manual goniometer to ensure the reliability and consistency. All participants were examined as follows: (i) During arm 0° abduction, the participants stayed fully relax, and upper arm rested gently against the chest wall at 0° abduction angle, with elbows at 90 degrees of flexion and upper arm in the same plane of chest wall; (ii) During arm 90° active abduction, arm was actively positioned at 90° abduction angle, with the elbow fully extended and palms down and arm in the same plane of chest wall. They were asked to maintain each arm abduction position for thirty seconds to cooperate with our inspection and obtain stable SWE images. To avoid muscle fatigue, the participants were asked to relax for 3 min after the examination of every arm abduction position.

**Figure 1:**
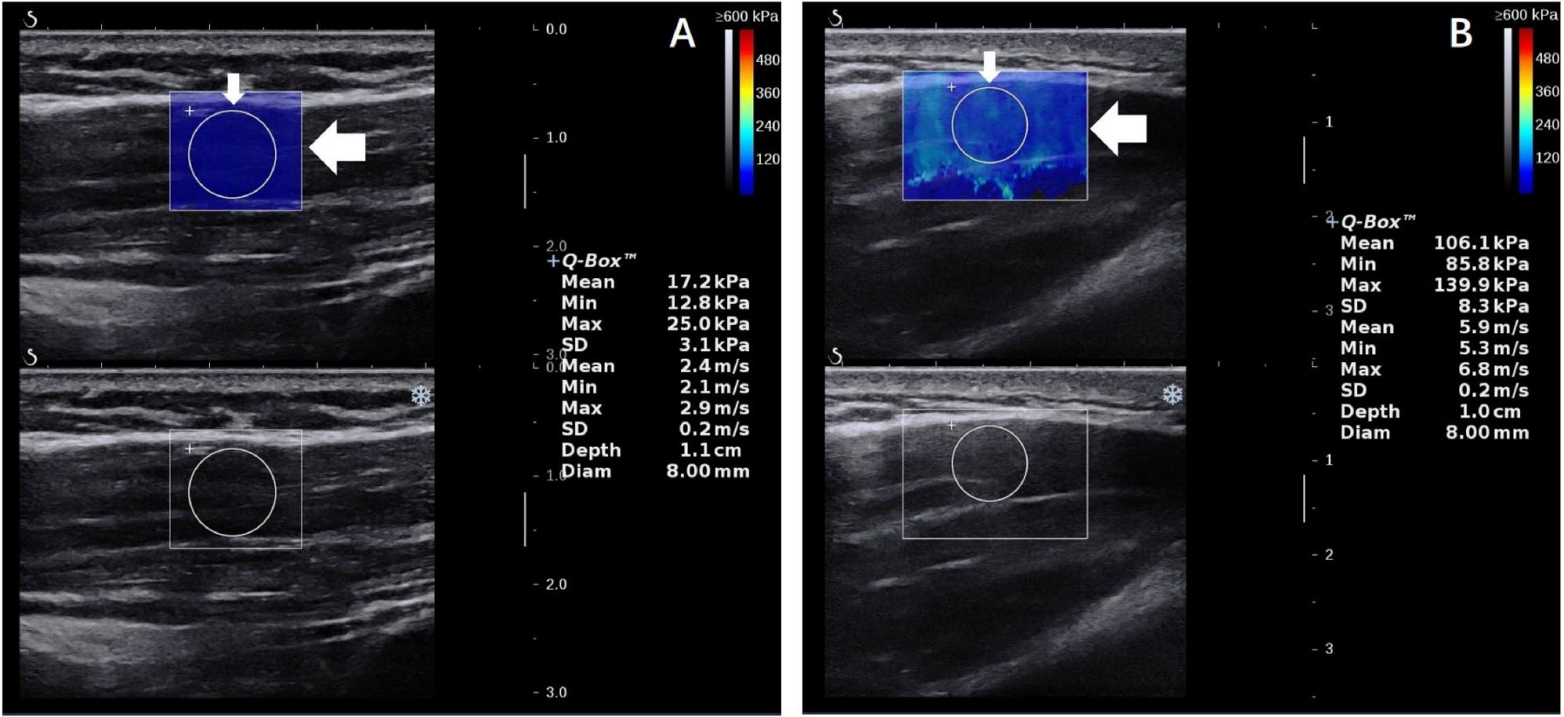
SWE (top panel) ultrasound images of the MD. A color map of MD elasticity is shown in the round Q-box (thin arrow) and square ROI (thick arrow). *MD* middle deltoid, *SWE* shear wave elastography, *SWV* mean shear wave velocity.

Both SWV in SWE images and MD thickness were measured three times in each arm abduction position by the same investigator, averaged, and expressed in m/s and millimeter, respectively. Figure 1 illustrates that the SWV values for the Q-box area expressed in m/s at R0° and R90°. SWV was measured in a round area called Q-box. The size of Q-box was fixed as a diameter of 8 mm in our study, and Q-box positioning was within the square region of interest (ROI) colormap, avoiding the deep and superficial fascia of the MD. The size of the ROI was adjusted according to the thickness of the target MD. The site of the target MD was fixed at the midpoint level of the MD muscle belly. Specifically, skin surface landmark was at the midpoint from deltoid tuberosity to the midpoint of the acromion^18^. In this region, muscle fibers of the targeted MD were basically parallel and the ROI colormap was stable in our study.

### Statistical analysis

The data were analyzed using SPSS v21.0 software (IBM, Armonk, NY, USA). All measurement data were conformed to normal distribution. Goodness of fit (R^2^) was obtained by linear regression analysis to determine the intra- and inter-operator reproducibility. The intraclass correlation coefficient (ICC) was calculated. Paired t-test between different arm abduction, Independent sample t-test for a binary variable (gender), and Pearson correlation test for continuous variables (MD thickness, BMI, age) were used. The lower and upper limits of reference ranges of normal MD elasticity were mean SWV-1.96 × SD (the standard deviation of mean SWV) and mean SWV + 1.96 × SD, respectively^17^. Two-sided *P* < 0.05 was considered statistically significant.

## Results

### Characteristics of healthy participants

The characteristics of 70 healthy subjects were summarized in Table 1. There were 35 males and 35 females in total subjects. The mean age ± SD was 42.10 ± 11.90 years (range, 19–70 years). The mean BMI was 23.20 ± 2.50 kg/m^2^. The mean MD thickness was 14.60 ± 0.61 mm at L0°, 15.10 ± 0.51 mm at R0°, 20.60 ± 1.42 mm at L90°, 21.80 ± 1.40 mm at R90°, respectively.

**Table 1:** Basic characteristics of 70 healthy participants enrolled in this study.

Characteristics	Healthy participants
Number	70
Female	35 (50.0%)
Male	35 (50.0%)
**Age (y)**	
All	42.10 ± 11.90
Female	45.90 ± 12.30
Male	38.30 ± 11.06
**BMI, kg/m**^**2**^	
All	23.20 ± 2.50
Female	22.90 ± 2.23
Male	23.50 ± 2.5 9
**MD thickness (mm)**	
L0°	14.60 ± 0.61
R0°	15.10 ± 0.51
L90°	20.60 ± 1.42
R90°	21.80 ± 1.40

Data presented as mean ± SD and number (percent) where applicable.

*MD* middle deltoid, *mm* millimeter, *SD* Standard deviation, *BMI* body mass index, *L0°* left arm 0° abduction, *R0°* right arm 0° abduction, *L90°* left arm 90° active abduction, *R90°* right arm 90° active abduction.

### Reliability study

Tables 2 and 3 showed that ICC and R^2^ values reflected the excellent intra- and inter- operator reproducibility of the MD elasticity measurement during different arm abduction in our study. The inter- and intra-operator reproducibility of the mean SWV of the MD at bilateral arm 0° abduction and 90° active abduction were listed in Tables 2 and 3. The ICC values showed the inter-operator reproducibility at L0° (ICC = 0.89), R0° (ICC = 0.92), L90° (ICC = 0.91), and R90° (ICC = 0.93); and the intra-operator reproducibility at L0° (ICC = 0.85), R0° (ICC = 0.91), L90° (ICC = 0.95), and R90° (ICC = 0.96).

**Table 2:** Intra-operator reproducibility of mean shear wave velocity of the MD at various arm abduction.

Shoulder abduction	SWV, m/s		ICC	*P*	R^2^
	A1	A2			
L0°	3.48 ± 0.28	3.32 ± 0.56	0.85	< 0.01	0.654
R0°	3.66 ± 0.32	3.59 ± 0.29	0.91	< 0.01	0.929
L90°	5.58 ± 0.41	5.53 ± 0.72	0.95	< 0.01	0.932
R90°	5.93 ± 0.49	5.91 ± 0.66	0.96	< 0.01	0.971

*A1* Operator A first measurement, *A2* operator A second measurement, *SWV* mean shear wave velocity, *ICC* intra-class correlation coefficient, R^2^ = goodness of fit. Data are presented as mean ± standard deviation where applicable.

**Table 3:** Inter-operator reproducibility of mean shear wave velocity of the MD at various arm abduction.

Shoulder abduction	SWV, m/s		ICC	*P*	R^2^
	Operator A	Operator B			
L0°	3.48 ± 0.28	3.60 ± 0.39	0.89	< 0.01	0.755
R0°	3.66 ± 0.32	3.57 ± 0.78	0.92	< 0.01	0.862
L90°	5.58 ± 0.41	5.52 ± 0.61	0.91	< 0.01	0.902
R90°	5.93 ± 0.49	5.90 ± 0.52	0.93	< 0.01	0.965

*SWV* mean shear wave velocity, *ICC* intra-class correlation coefficient, R^2^ = goodness of fit. Data are presented as mean ± standard deviation where applicable.

### Influence of different arm abduction

**Figure 2:**
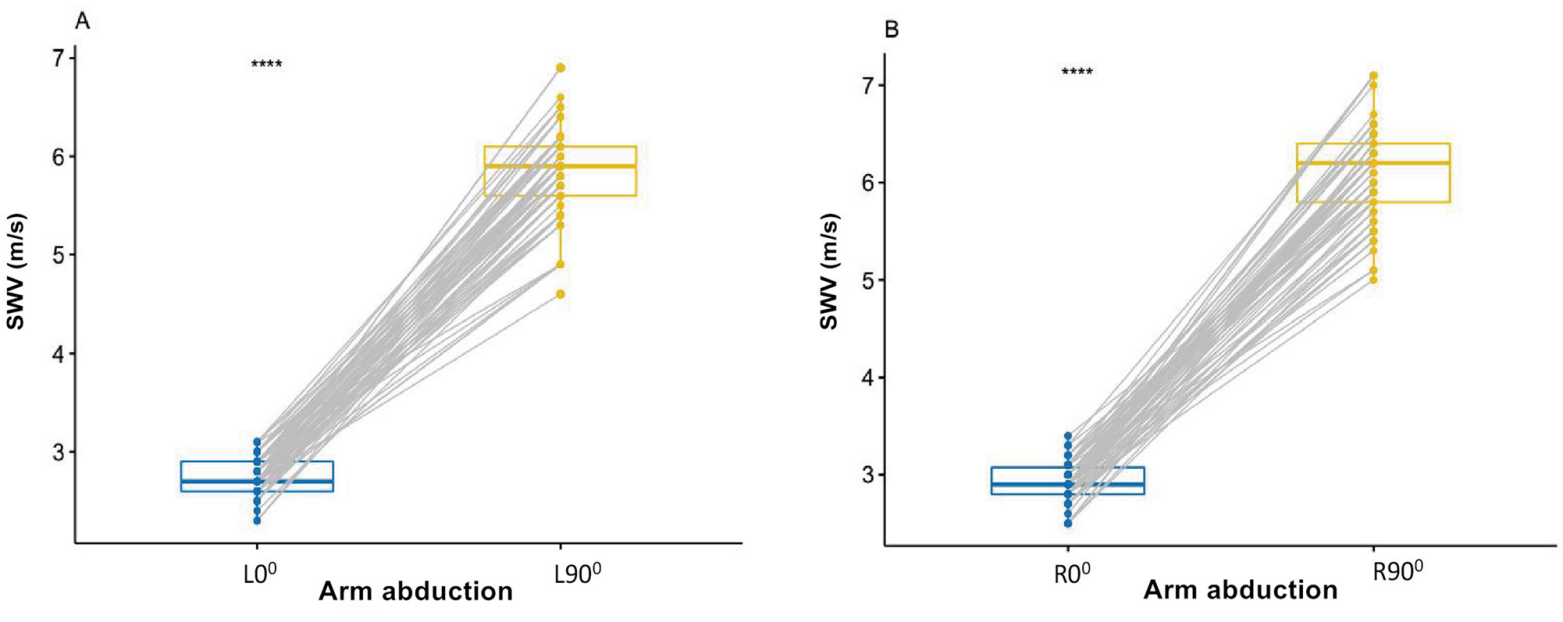
Box-and-whisker plots of the SWV in healthy participants using SWE between L0° and L90° (**A**), between R0° and R90° (**B**). (**A**). Difference of the SWV between L0° and L90°, (**B**) Difference of the SWV between R0° and R90°; ****p < .0001. *SWV* mean shear wave velocity.

**Figure 3:**
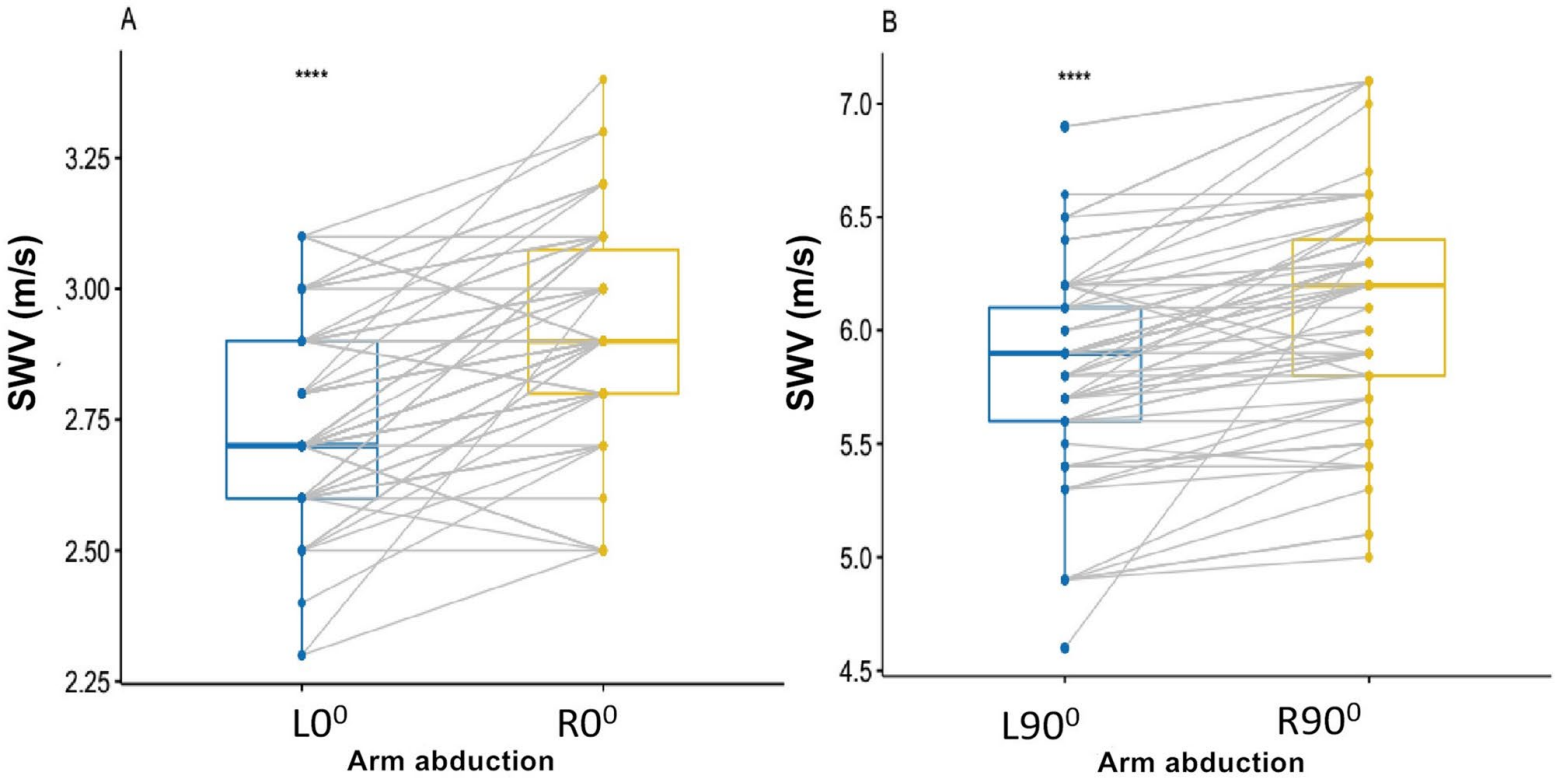
Box-and-whisker plots of the SWV in healthy participants with SWE between L0° and R0° (**A**), between L90° and R90° (**B**). ****p < .0001. *SWV* mean shear wave velocity.

SWV was significantly higher at L90° than L0° (*P* < 0.0001; Fig. 2A), and higher at R90° than R0° (*P* < 0.0001; Fig. 2B). SWV was also significantly higher at R0° than L0° (*P* < 0.0001; Fig. 3A), and higher at R90° than L90° (*P* < 0.0001; Fig. 3B).

### Influence of gender

**Figure 4:**
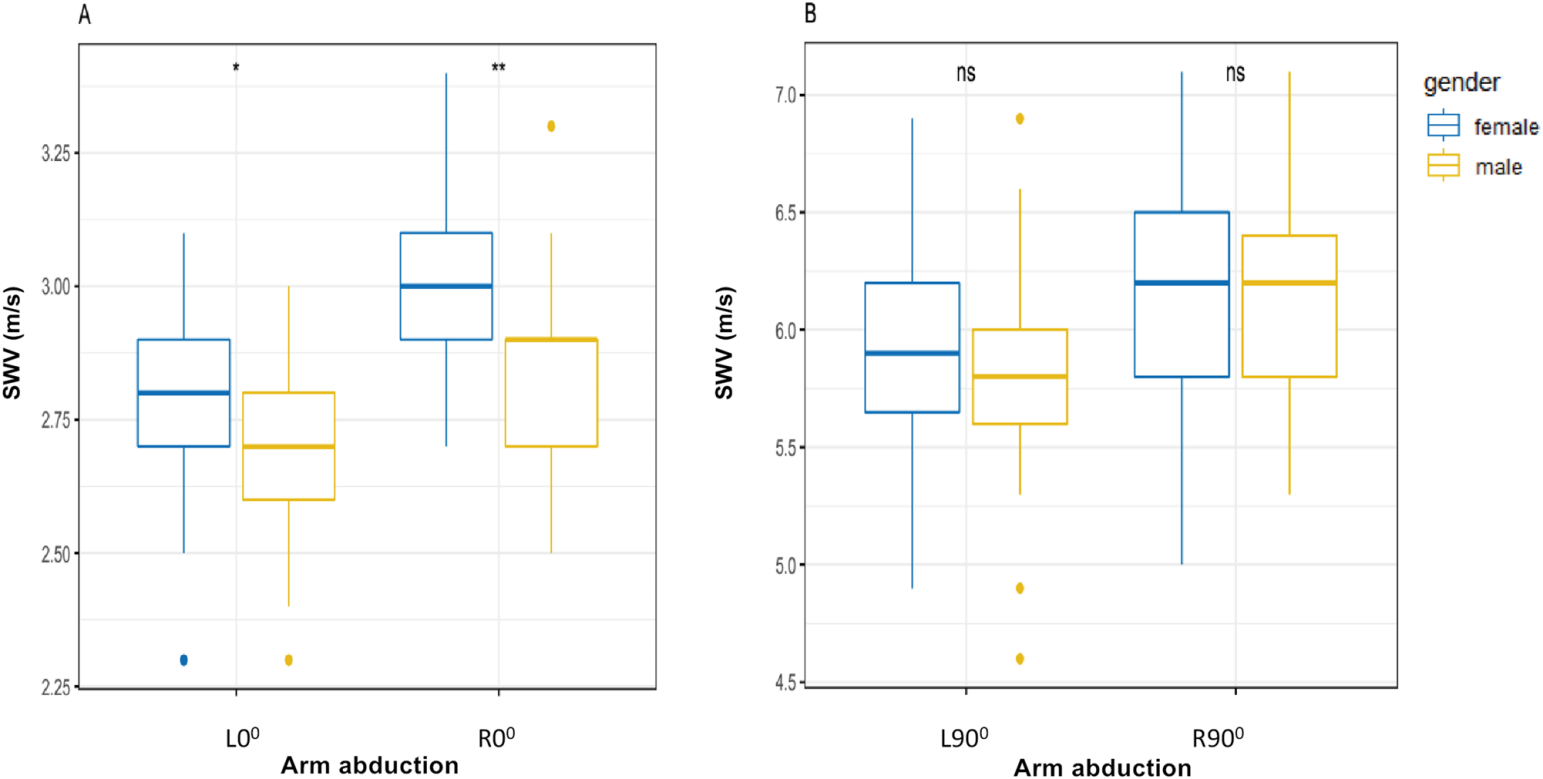
Box plots of the SWV in healthy participants between different gender at L0°, R0°, L90° and R90° respectively. (**A**) Difference of the SWV between female and male at L0° and R0°; (**B**) Difference of the SWV between females and males at L90° and R90°; ns, no significance; *p < .05; **p < .01. *SWV* mean shear wave velocity.

SWV at both L90° (*P* = 0.58, > 0.05) and R90° (*P* = 0.71, > 0.05) were not significantly different between males and females (Fig. 4B), while SWV at both L0° (*P* < 0.05) and R0° (*P* < 0.01) were higher in males than in females (Fig. 4A).

### Influence of MD thickness

**Figure 5:**
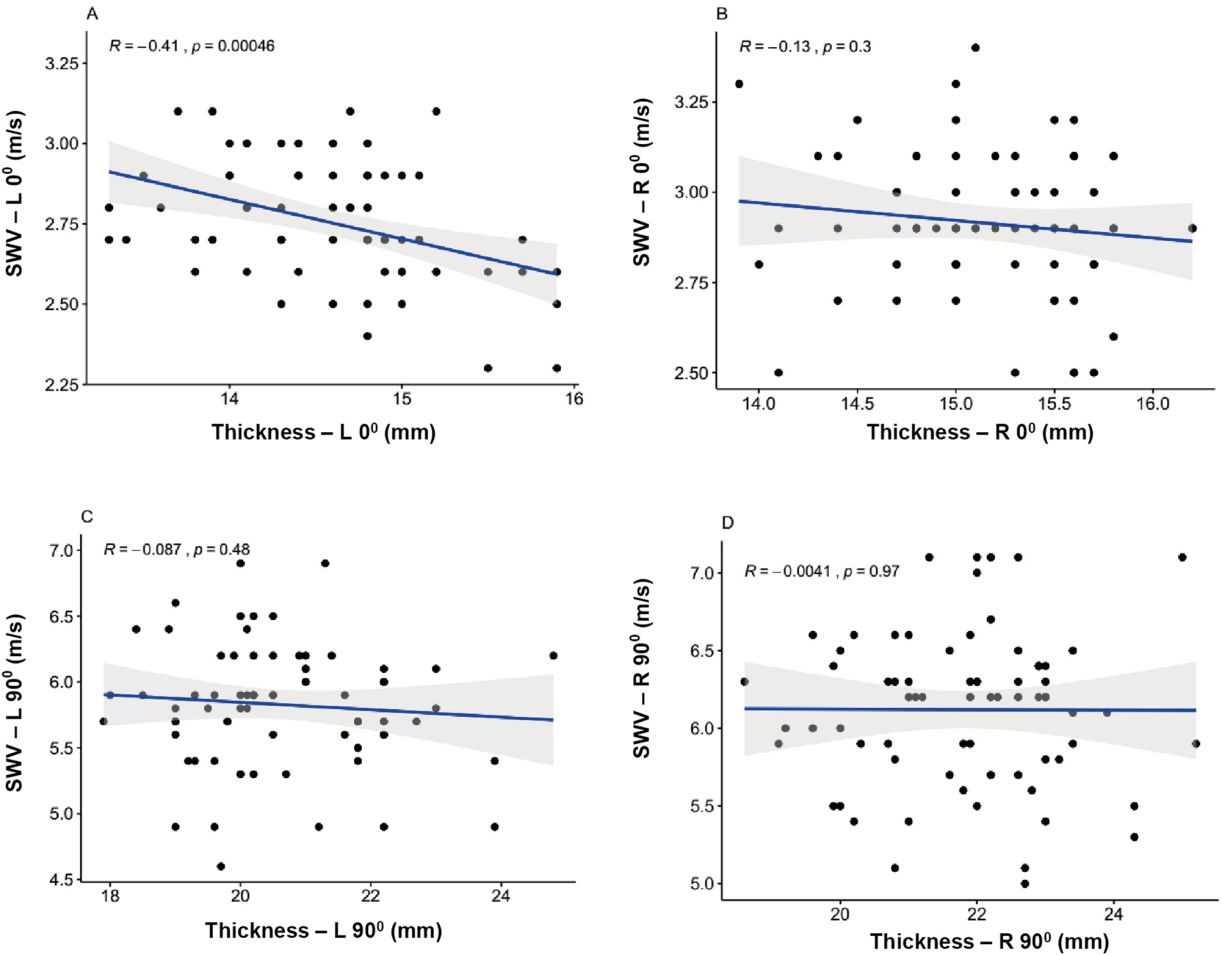
Scatterplots of MD thickness and SWV in healthy participants at L0° (**A**), R0° (**B**), L90° (**C**), and R90° (**D**). *MD* middle deltoid; *SWV* mean shear wave velocity.

MD thickness was not related to SWV at R0° (R = − 0.13, *P* = 0.3, > 0.05), L90° (R = − 0.087, *P* = 0.48, > 0.05) and R90° (R = − 0.004, *P* = 0.97, > 0.05) (Fig. 5B–D). Mild positive correlation can be neglected at L0° (R = − 0.41, *P* < 0.01) (Fig. 5A) between MD thickness and SWV.

### Influence of age

**Figure 6:**
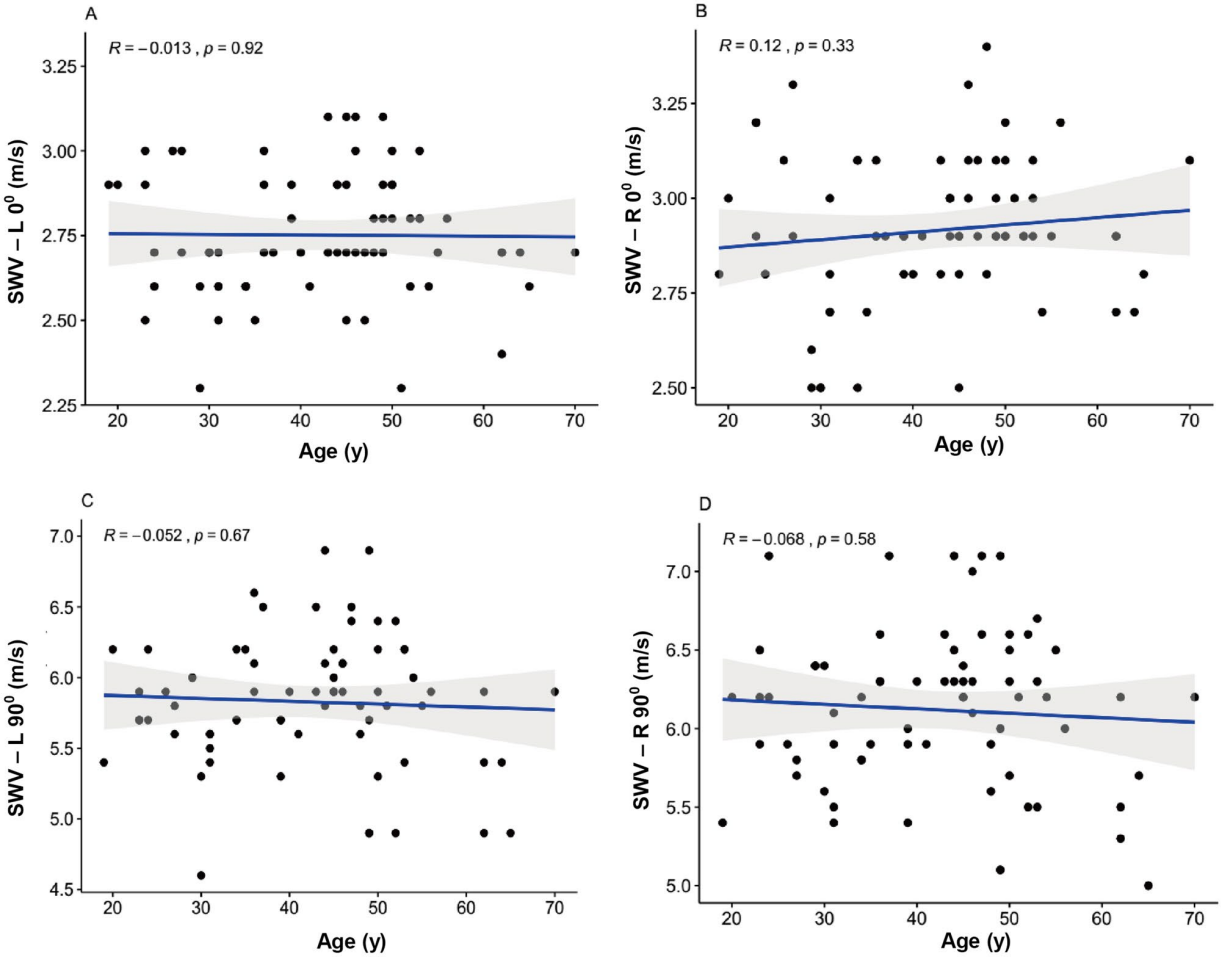
Scatterplots of age and SWV in healthy participants at L0° (**A**), R0° (**B**), L90° (**C**), and R90° (**D**). *SWV* mean shear wave velocity.

There was no significant correlation between SWV and age at L0° (R = − 0.013, *P* = 0.92, > 0.05), R0° (R = 0.12, *P* = 0.33, > 0.05), L90° (R = − 0.052, *P* = 0.67, > 0.05) and R90° (R = − 0.068, *P* = 0.58, > 0.05) (Fig. 6A–D).

### Influence of BMI

**Figure 7:**
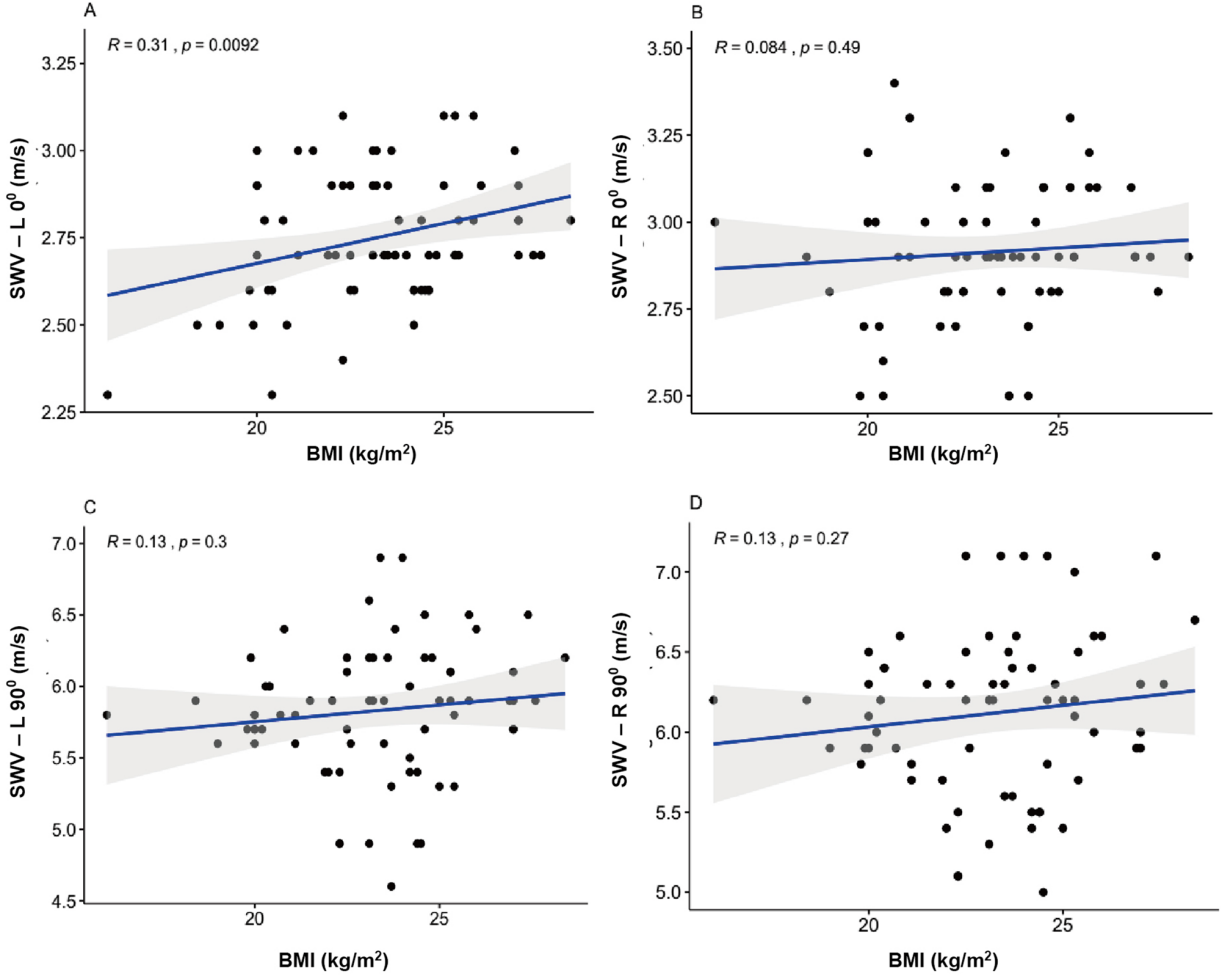
Scatterplots of BMI and SWV in healthy participants at L0° (**A**), R0° (**B**), L90° (**C**), and R90° (**D**). *SWV* mean shear wave velocity, *BMI* body mass index.

The BMI was not significantly correlated with SWV at R0° (R = 0.084, *P* = 0.49, > 0.05), L90° (R = 0.13, *P* = 0.3, > 0.05) and R90° (R = 0.13, *P* = 0.27, > 0.05) (Fig. 7B–D). Slight statistically positive correlation can be ignored at L0° (R = 0.31, *P* = 0.0092, < 0.05) (Fig. 7A) between SWV and BMI.

### Reference ranges of normal MD elasticity

The reference ranges of normal MD elasticity were 2.4–3.1 m/s in males and 2.2–2.9 m/s in females at L0° and 2.5–3.3 m/s in males and 2.4–3.2 m/s in females at R0°, and were 4.9–6.7 m/s at L90°, 5.2–7.1 m/s at R90° for both males and females. Normal reference ranges of the MD elasticity at L0°, R0°, L90° and R90° were listed in Table 4.

**Table 4:** Reference ranges of MD elasticity in healthy participants by gender at various arm abduction.

Shoulder abduction	Gender	SWV, m/s		
		Mean ± SD	95% CI	Reference range
L0°	Male	2.70 ± 0.18	2.7–2.8	2.4–3.1
	Female	2.59 ± 0.16	2.5–2.6	2.2–2.9
R0°	Male	2.90 ± 0.21	2.9–3.0	2.5–3.3
	Female	2.80 ± 0.17	2.8–2.9	2.4–3.2
L90°	Male/Female	5.80 ± 0.46	5.7–5.9	4.9–6.7
R90°	Male/Female	6.10 ± 0.50	6.0–6.2	5.2–7.1

*SD* Standard deviation, *MD* middle deltoid, *SWV* mean shear wave velocity, *CI* confidence interval. *L0°* left arm 0° abduction, *R0°* right arm 0° abduction, *L90°* left arm 90° active abduction, *R90°* right arm 90° active abduction.

## Discussion

The major finding of the present study was that MD elasticity was higher on the right arm than on the left one at both 0° and 90° active abduction (all *P* < 0.0001). It may be because right arm is more frequently used. Moreover, MD elasticity was higher at R90° than R0° (*P* < 0.0001), at L90° than L0° (*P* < 0.0001). These results are similar to our previous study on normal upper trapezius muscle elasticity^18^. It may be due to intense contraction of the MD during arm 90° active abduction. During arm active abduction, the MD produces active tension produced by MD muscle contraction, thereby increasing MD elasticity^3,4^. In particular, gender had affected MD elasticity at both L0° (*P* < 0.05) and R0° (*P* < 0.01), maybe indicating there is significant difference in the MD muscle size and component at arm 0° abduction between different gender^19–22^. Previous study showed that different elastin collagen of muscle fibers may result in the change in muscle elasticity between male and female^22–25^. We also found excellent intra- and inter-operator reliability using SWE to measure MD elasticity at L0°, R0°, L90°, and R90°^13,26–28^. The reasons for excellent inter- and intra-operator reproducibility include standardized examining method and skilled sonographers, the basically parallel muscle fibers of the target MD in our study. It indicates that SWE is a reliable and feasible technique for assessing MD elasticity.

The purpose of this study was to preliminarily set up the normal reference ranges of MD elasticity during 0° arm abduction and 90° active arm abduction for bilateral examination using SWE. Although it was a preliminary exploratory research, these reference ranges may serve as quantitative baseline measurements for assessment of normal MD elasticity in the future. Moreover, the MD elasticity using SWE has important clinical significance for reverse shoulder arthroplasty. It may provide useful information of the MD mechanical properties during treatment of reverse shoulder arthroplasty.

This study has some features from several perspectives. The new technique called SWE can quantitatively reflect biomechanics changes of muscles contractility. Increased muscle elasticity during arm active abduction, resulting from muscle contraction, is an especially exciting area for clinical integration of ultrasound elastography^28–30^. Firstly, it may be the first study to quantitatively assess normal MD elasticity by measuring the SWV during different arm active abduction. One the one hand, many previous studies have been about the effects of different head postures on neck or shoulder muscles^31–33^. Meanwhile, reviewing the literature, few studies have done on muscle elasticity at different active abduction of bilateral shoulder. On the other hand, elastic parameter of most previous researches on assessing muscle elasticity by SWE have almost been Young's modulus. Nevertheless, our study assessed MD elasticity according to the elastic parameter SWV. Secondly, this work may be the first study to evaluate impacting factors affecting MD elasticity by SWE, including age, gender, BMI and MD thickness. No previous studies have analyzed all these potential factors as we did. Thirdly and most importantly, the clinical significance of establishing reference ranges of normal MD elasticity lies in providing preliminary exploration for serving as the baseline measurement of normal MD elasticity. Using this new ultrasound elastography technique SWE, the reference ranges of normal MD elasticity have hardly been studied before^33–35^. If reference ranges of normal MD elasticity can be generalized to a larger healthy population in the future, the clinical significance will be even greater. Our study may also provide new ideas for evaluation of muscle elasticity under contraction state using SWE.

There are some limitations in our research. The number of participants is a small sample as a preliminary study. In order to extend the reference ranges of normal MD elasticity to the larger healthy population, we will increase sample size and conduct multi-center studies in the next step. To provide baseline measurements for diagnosis of the diseased MD muscle, the diseased population of the MD should be studied in the next step. Moreover, considering impact of multiple factors, we will use linear mixed model for investigate the combined effects of confounding factors such as age, gender and so on in the further studies.

In conclusion, our results suggest that the normal MD elasticity at L0°, R0°, L90°, and R90° with SWE are obviously different. These established reference ranges may serve as quantitative baseline measurements for assessment of normal MD elasticity in the future. Moreover, SWE can be used to assess muscle activation by measuring the change in muscle elasticity at various arm abduction.

## Data Availability

The datasets used and analyzed during the current study are available from the corresponding author on reasonable request.

## References

[1] Hatta T (2016). Quantified mechanical properties of the deltoid muscle using the shear wave elastography: potential implications for reverse shoulder arthroplasty. PLoS ONE.

[2] Walker DR, Struk AM, Matsuki K, Wright TW, Banks SA (2016). How do deltoid muscle moment arms change after reverse total shoulder arthroplasty?. J. Shoulder Elbow Surg..

[3] Hatta T (2015). Quantitative assessment of rotator cuff muscle elasticity: reliability and feasibility of shear wave elastography. J. Biomech..

[4] Sakoma Y (2011). Anatomical and functional segments of the deltoid muscle. J. Anat..

[5] Akagi R, Kusama S (2015). Comparison between neck and shoulder stiffness determined by shear wave ultrasound elastography and a muscle hardness meter. Ultrasound Med. Biol..

[6] Mayoux-Benhamou MA, Revel M, Vallee C (1997). Selective electromyography of dorsal neck muscles in humans. Exp. Brain. Res..

[7] Gennisson JL, Cornu C, Catheline S, Fink M, Portero P (2005). Human muscle hardness assessment during incremental isometric contraction using transient elastography. J. Biomech..

[8] Bizzini M, Mannion AF (2003). Reliability of a new, hand-held device for assessing skeletal muscle stiffness. Clin. Biomech..

[9] Kuo YC, Hsieh LF (2019). Validity of cyriax's functional examination for diagnosing shoulder pain: a diagnostic accuracy study. J. Manip. Physiol. Ther..

[10] Eby SF (2013). Validation of shear wave elastography in skeletal muscle. J. Biomech..

[11] Cortez CD (2016). Ultrasound shear wave velocity in skeletal muscle: a reproducibility study. Diagn. Interv. Imaging.

[12] Alfuraih AM (2007). An investigation into the variability between different shear wave elastography systems in muscle. Med. Ultrason..

[13] Kim K, Hwang HJ, Kim SG, Lee JH, Jeong WK (2018). Can shoulder muscle activity be evaluated with ultrasound shear wave elastography?. Clin. Orthop. Relat. Res..

[14] Miyamoto N, Hirata K, Kimura N, Miyamoto-Mikami E (2018). Contributions of hamstring stiffness to straight-leg-raise and sit-and-reach test scores. Int. J. Sports Med..

[15] Bercoff J, Tanter M, Fink M (2004). Supersonic shear imaging: a new technique for soft tissue elasticity mapping. IEEE. Trans. Ultrason. Ferroelectr. Freq. Control.

[16] Hug F, Tucker K, Gennisson JL, Tanter M, Nordez A (2015). Elastography for muscle biomechanics: toward the estimation of individual muscle force. Exerc. Sport. Sci. Rev..

[17] Chino K, Kawakami Y, Takahashi H (2017). Tissue elasticity of in vivo skeletal muscles measured in the transverse and longitudinal planes using shear wave elastography. Clin. Physiol. Funct. Imaging.

[18] Wang L, Xiang X, Zhu B, Qiu L (2020). Determination of reference ranges for normal upper trapezius elasticity during different shoulder abduction using shear wave elastography: a preliminary study. Sci. Rep..

[19] Leong HT, Ng GY, Leung VY, Fu SN (2013). Quantitative estimation of muscle shear elastic modulus of the upper trapezius with supersonic shear imaging during arm positioning. PLoS ONE.

[20] Lee H, Kim K, Lee Y (2020). Development of stiffness measurement program using color mapping in shear wave elastography. Diagnostics.

[21] Shinohara M, Sabra K, Gennisson JL, Fink M, Tanter M (2010). Realtime visualization of muscle stiffness distribution with ultrasound shear wave imaging during muscle contraction. Muscle Nerve.

[22] Bouillard K, Nordez A, Hug F (2011). Estimation of individual muscle force using elastography. PLoS ONE.

[23] Janssen I, Heymsfield SB, Wang ZM, Ross R (2000). Skeletal muscle mass and distribution in 468 men and women aged 18–88 yr. J. Appl. Physiol..

[24] Whittaker JL, Stokes M (2011). Ultrasound imaging and muscle function. J. Orthop. Sports Phys. Ther..

[25] Haizlip KM, Harrison BC, Leinwand LA (2015). Sex-based differences in skeletal muscle kinetics and fiber-type composition. Physiology.

[26] Itoigawa Y (2015). Feasibility assessment of shear wave elastography to rotator cuff muscle. Clin. Anat..

[27] Yoshitake Y, Takai Y, Kanehisa H, Shinohara M (2014). Muscle shear modulus measured with ultrasound shear-wave elastography across a wide range of contraction intensity. Muscle Nerve.

[28] Ewertsen C, Carlsen J, Perveez MA, Schytz H (2018). Reference values for shear wave elastography of neck and shoulder muscles in healthy individuals. Ultrasound Int. Open.

[29] Drakonaki EE, Allen GM, Wilson DJ (2012). Ultrasound elastography for musculoskeletal applications. Br. J. Radiol..

[30] Lee Y, Kim M, Lee H (2021). The measurement of stiffness for major muscles with shear wave elastography and myoton: a quantitative analysis study. Diagnostics.

[31] Goodarzi F, Rahnama L, Karimi N, Baghi R, Jaberzadeh S (2018). The effects of forward head posture on neck extensor muscle thickness: an ultrasonographic study. J. Manip. Physiol Ther..

[32] Shin YJ, Kim WH, Kim SG (2017). Correlations among visual analogue scale, neck disability index, shoulder joint range of motion, and muscle strength in young women with forward head posture. J. Exerc. Rehabil..

[33] Sadeghi S, Johnson M, Bader DA, Cortes DH (2019). The shear modulus of lower-leg muscles correlates to intramuscular pressure. J. Biomech..

[34] Dubois G (2015). Reliable protocol for shear wave elastography of lower limb muscles at rest and during passive stretching. Ultrasound Med. Biol..

[35] Schmalzl J, Fenwick A, Boehm D, Gilbert F (2017). The application of ultrasound elastography in the shoulder. J. Shoulder Elbow Surg..

